# Supplementary material for: Stage II of Chronic Kidney Disease—A Tipping Point in Disease Progression?
Source: Biomedicines. 2022 Jun 27;10(7):1522. doi: 10.3390/biomedicines10071522 (PMC9313233; doi:10.3390/biomedicines10071522)
Supplement: Supplementary file 1 [file biomedicines-10-01522-s001.zip › biomedicines-1776432-supplementary.pdf]

Supplementary Table S1. Summary information regarding identified proteins per group, as well as the total number of identified proteins.

	Healthy controls	CKD 1	CKD 2	CKD 3	CKD 4	CKD 5
Participants per pool	15	15	15	15	15	15
Identified proteins	371	356	352	381	374	389
Unique outliers	5	1	7	0	1	5
Total proteins	453					

CKD – chronic kidney disease

Supplementary Table S2. The table shows all biological pathways to which the identified proteins in each group showed a statistically significant relation by the FunRich 3.1.3. software. The table values represent the percentage of identified proteins that take part in the identified pathway in the respective study group. Statistically significant pathways were manually curated and presented in the Results section of the paper.

PATHWAY	CONTROLS	CKD 1	CKD 2	CKD 3	CKD 4	CKD 5
Beta2 integrin cell surface interactions	6.1403509	5.504587	6.422018	5.982906	5.932203	5.833333
Beta3 integrin cell surface interactions	6.1403509	6.422018	6.422018	5.982906	5.932203	5.833333
Chylomicron-mediated lipid transport	6.1403509	6.422018	6.422018	5.982906	5.932203	6.666667
Classical antibody-mediated complement activation	4.3859649	4.587156	4.587156	4.273504	4.237288	4.166667
Common Pathway	10.526316	10.09174	11.00917	10.25641	10.16949	10
Complement cascade	13.157895	13.76147	13.76147	12.82051	12.71186	12.5
Creation of C4 and C2 activators	5.2631579	5.504587	5.504587	5.128205	5.084746	5
Diabetes pathways	13.157895	12.84404	13.76147	12.82051	12.71186	12.5
Dissolution of Fibrin Clot	3.5087719	3.669725	3.669725	3.418803	3.389831	3.333333
Epithelial-to-mesenchymal transition	14.035088	13.76147	13.76147	13.67521	13.55932	13.33333
Formation of Fibrin Clot (Clotting Cascade)	18.421053	18.34862	18.34862	17.94872	17.79661	17.5
Gamma-carboxylation of protein precursors	4.3859649	3.669725	4.587156	4.273504	4.237288	4.166667
Gamma-carboxylation, transport, and amino-terminal cleavage of proteins	4.3859649	0	4.587156	4.273504	4.237288	4.166667
HDL-mediated lipid transport	6.1403509	6.422018	6.422018	5.982906	5.932203	5.833333
Hemostasis	28.070175	28.44037	28.44037	27.35043	27.11864	26.66667
Immune System	21.052632	0	22.01835	21.36752	21.18644	20.83333
Initial triggering of complement	7.8947368	8.256881	8.256881	7.692308	7.627119	7.5
Innate Immune System	14.035088	14.6789	15.59633	14.52991	14.40678	14.16667
Integrin cell surface interactions	7.8947368	8.256881	7.33945	7.692308	0	7.5
Integrins in angiogenesis	0	0	0	0	6.779661	0
Intrinsic Pathway	10.526316	11.00917	11.00917	10.25641	10.16949	10
Lipid digestion, mobilization, and transport	8.7719298	9.174312	9.174312	8.547009	8.474576	9.166667
Lipoprotein metabolism	8.7719298	9.174312	9.174312	8.547009	8.474576	9.166667
Platelet degranulation	5.2631579	5.504587	0	5.128205	5.084746	5
Post-translational modification: gamma carboxylation and hypusine formation	4.3859649	0	4.587156	4.273504	4.237288	4.166667
Regulation of Insulin-like Growth Factor (IGF) Activity by Insulin-like Growth Factor Binding Proteins (IGFBPs)	7.8947368	8.256881	8.256881	7.692308	7.627119	7.5
Removal of aminoterminal propeptides from gamma-carboxylated proteins	4.3859649	3.669725	4.587156	4.273504	4.237288	4.166667
Terminal pathway of complement	5.2631579	5.504587	5.504587	5.128205	5.084746	5
Transport of gamma-carboxylated protein precursors from the endoplasmic reticulum to the Golgi apparatus	4.3859649	3.669725	4.587156	4.273504	4.237288	4.166667
